# Pharmacokinetics of nalbuphine hydrochloride extended release tablets in hemodialysis patients with exploratory effect on pruritus

**DOI:** 10.1186/s12882-015-0043-3

**Published:** 2015-04-08

**Authors:** Amale Hawi, Harry Alcorn, Jolene Berg, Carey Hines, Howard Hait, Thomas Sciascia

**Affiliations:** A Hawi Consulting, Ridgefield, CT USA; DaVita Clinical Research, Minneapolis, MN USA; PPD, Richmond, VA USA; Edenridge Associates LLC, Wilmington, DE USA; Trevi Therapeutics, 195 Church Street, 14th Floor, New Haven, CT 06510 USA

**Keywords:** ESRD, Hemodialysis, Opioid, Pharmacokinetics, Pruritus, Safety, Nalbuphine, Itch

## Abstract

**Background:**

Uremic pruritus is a common and deleterious condition among hemodialysis (HD) patients. Central gating of μ/κ opiate circuitry plays an important role in mediating and countering pruritogenic sensation. The objective of this study was to assess the safety and pharmacokinetics (PK) of the mixed μ-antagonist/κ-agonist nalbuphine, administered orally as nalbuphine HCl extended release (ER) tablets in HD patients, and explore its effect on pruritus.

**Methods:**

In this open-label multiple escalating dose study, 15 HD patients with pruritus and 9 matched healthy subjects were enrolled. Nalbuphine HCl ER dose was escalated from 30 mg QD to 240 mg BID over 15 days. A full PK profile was obtained under dialysis and non-dialysis conditions as a function of dose. Clearance during dialysis was determined by sampling dialysate and arterial/venous blood during dialysis. Pruritus severity was assessed twice daily using a Visual Analog Scale (VAS). Safety monitoring included extensive monitoring of EKG, blood pressure, and pulse oximetry.

**Results:**

In HD patients, nalbuphine concentration peaked within 4–9 hours and attained steady state within 2–3 days, with no significant accumulation. Mean half-life was 14.2 hours, mean C_max_ and AUC_tau_ ranged between 13 and 83 ng/mL and 118 and 761 ng∙h/mL, respectively, with exposure increasing in a nearly dose-proportional fashion. Exposure in HD patients was about 2-fold higher than in healthy subjects. There was no meaningful difference between exposure on dialysis and non-dialysis days with 1% or less of the dose removed by dialysis. Nalbuphine suppressed itch in a dose-dependent manner, reducing mean VAS score from 4.0 to 1.2 at 180 mg and 0.4 at 240 mg.

**Conclusions:**

Nalbuphine HCl ER tablets can be safely administered to HD patients without dose adjustment up to 240 mg BID and may hold promise in treating uremic pruritus.

**Electronic supplementary material:**

The online version of this article (doi:10.1186/s12882-015-0043-3) contains supplementary material, which is available to authorized users.

## Background

Uremic pruritus is an itch disorder associated with end-stage renal disease (ESRD) that can be severe and debilitating. In its most severe form, uremic pruritus is associated with significant deleterious impairments of patient quality of life, including depression and disruption of sleep [1,2]. A 17% increase in mortality rate (p < 0.001), attributed to sleep disturbances, is associated with moderate to severe pruritus [2,3]. Uremic pruritus is independent of gender, age, ethnicity, type of dialysis, and the etiology of the underlying renal disease [3,4]. Among the factors causing pruritus in ESRD patients are accumulation of uremic toxins, systemic inflammation, cutaneous xerosis, and common comorbidities, e.g. diabetes mellitus and viral hepatitis (4). Currently, there are no approved treatments in the United States or Europe. Uremic pruritus is typically treated with creams, antihistamines, ultraviolet radiation, and the off-label use of various drugs, including opioids, with limited success [4-6]. To date, uremic pruritus remains an unresolved problem with renal transplantation being the only effective treatment [7,8].

Understanding of the pathogenesis of uremic pruritus has evolved considerably over the past decade as the underlying pathophysiology of pruritus sensation and itch are more rigorously investigated. A mechanistic hypothesis related to peripheral neuropathic changes and central nervous system pathobiology along with evidence for cutaneous microinflammation has recently emerged [6,9]. As such, pruritus and pain are believed to share many neurophysiological processes, yet distinct pathways [4]. While central μ-opioid receptor agonism induces itching that can be abolished with μ-antagonists, κ-opioid receptor agonism inhibits the μ-receptor-mediated scratching [10,11]. Thus the central gating of μ/κ opiate circuitry could be important in countering pruritogenic sensation from a peripheral neurogenic inflammatory initiating event in uremic pruritus [12,13].

In addition to a potential neurophysiological mechanism connected to opioid receptor biology, uremic pruritus has been correlated to an imbalance between the endogenous opiate ligands beta-endorphin (μ-agonist) and dynorphin A (κ-agonist), resulting in an increased beta-endorphin to dynorphin A serum ratio in uremic patients compared to healthy volunteers [11]. Clinical study data support a role for opioid receptors in mediating itch processing in uremic pruritus: nalfurafine HCl, a pure κ-opioid receptor agonist, has been shown to reduce itch severity and sleep disturbances in uremic pruritus patients [14,15], while naltrexone, a μ-antagonist, has shown some beneficial effect in relieving uremic pruritus-associated itch, although with more limited success [16].

Nalbuphine is a mixed μ-antagonist/κ-agonist opioid drug [17], currently marketed as *Nalbuphine HCl for Injection* for use in the relief of moderate to severe pain [18]. In addition, nalbuphine has been shown to attenuate morphine-induced pruritus in a number of well-controlled, clinical studies [19-23]. More recently, nalbuphine was shown to significantly reduce Substance-P induced itch in a mouse model [24]. In view of its dual agonist/antagonist mechanism of action, nalbuphine may be effective at reducing pruritus by rebalancing opioid μ and κ neuronal activity.

An extended release (ER) nalbuphine solid oral dosage form was developed to facilitate drug administration and patient adherence. Understanding nalbuphine disposition following oral administration in the target HD patient population is critical as the effects of renal impairment on opioid clearance are variable [25-27]. This study was designed to assess the safety and pharmacokinetics (PK) of nalbuphine administered orally as nalbuphine HCl ER tablets in renally-impaired HD patients with pruritus following repeated escalating doses over a 6-fold dose range, and to determine whether nalbuphine is cleared by dialysis. In addition, the effect of nalbuphine on uremic pruritus was explored.

## Methods

This study was sponsored by Trevi Therapeutics and conducted in accordance with the Declaration of Helsinki. All aspects of the study were conducted in accordance with national, state, and local laws and regulations. The study was registered at clinicaltrials.gov (NCT02373215) and the study protocol, all amendments, and informed consent form (ICF) were reviewed and approved by the Investigator, clinic staff, and Institutional Review Board (Western Institutional Review Board, Olympia, WA). All patients provided written, signed informed consent prior to entering the study and before any study-related procedures were performed.

### Study drug and administration

Nalbuphine HCl ER tablets (30 mg) were provided by Trevi Therapeutics. Unless specified, doses were administered as multiples of 30-mg tablets to achieve the desired dose and with water (120 ml) 12 hours apart with food. All subjects received a renal/diabetic diet. For HD patients on dialysis days, the morning dose was administered no earlier than 6 hours and no later than 4 hours prior to dialysis; the evening dose was administered after the end of dialysis, 12 hours after the morning dose.

### Study subjects

Study subjects were 18–70 years of age. HD patients with Stage 5 chronic end-stage renal disease (ESRD) requiring dialysis reported at least mild intermittent pruritus at Screening (according to the clinical pharmacology unit Checklist of Common Symptoms of Dialysis Patients); had been undergoing dialysis 3x/week for at least 3 months with Kt/V >1.1 with no significant alteration in regimen within 2 weeks prior to Screening; and had hemoglobin > 9 g/dL at Screening. HD patients with alanine and/or aspartate aminotransferase concentration >2X the upper limit of normal range (ULN) and serum total bilirubin >1.8X ULN at Screening were excluded. Factors that may impact pruritus severity such as predialysis phosphate, urea and CRP levels were not examined in this study.

Healthy subjects were matched with HD patients for body mass index (BMI; within 15%), age (within 10 years), and gender. For all subjects, exclusion criteria included known hypersensitivity to nalbuphine or opioids; pregnancy or lactation; abnormal laboratory values considered clinically significant by the Investigator; and receipt of barbiturates, amphetamines, or opiates within 7 days prior to check-in.

### Study design

The study was an open-label, single site, multiple escalating dose study comprised of 2 cohorts. Per protocol, Cohort 1 consisted of 14 HD patients divided into four groups with 2, 2, 6 and 4 patients in each of Groups 1, 2, 3, and 4, respectively. Cohort 2 consisted of 8 healthy subjects. Subjects who discontinued study prior to reaching the final dose level (180 mg or 240 mg) were replaced. The targeted number of subjects is within the range of sample sizes used in similar Phase 1 clinical studies and is not based on a formal statistical power calculation.

Subjects received a single 30-mg dose on Day 1. Doses were subsequently escalated to twice daily (BID) 30 mg, 60 mg, 120 mg, 180 mg over 13 days or to 240 mg BID over 15 days (Cohort 1, Group 4 only). On the last treatment day, subjects received a single 180-mg or 240-mg dose in the morning. Subjects remained at each dose level for 2–3 days (minimum 4 consecutive doses) with dose escalation predicated on tolerability of the prior dose. Subjects remained in the clinic from Day −1 until discharge on Day 14 (~30 hours after last dose) or Day 17 (~54 hours after last dose for Cohort 1, Group 4). Subjects returned 5–7 days after discharge for safety follow-up evaluations. For subjects in Cohort 1, dialysis was conducted at approximately the same time on Days −1, 3, 5, 7, 10, 12, 14 (and Day 17 for Group 4) over 3–3.5 hours using a high-flux dialyzer with polysulfone membrane (Additional file 1).

**Figure 1:**
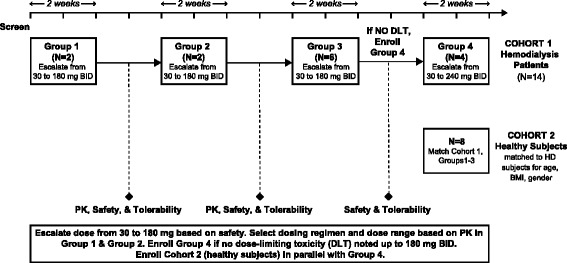
**Study schematic.**

Dosing of subjects in Cohort 1 Groups 1–4 was staggered to allow for an interim medical safety review and PK analysis. Since healthy subjects were matched to HD patients, dosing of Cohort 2 was not initiated until Cohort 1 Groups 1–3 were complete and the dosing regimen confirmed. All subjects in Cohort 2 were dosed concurrently. A study schematic is provided in Figure 1.

### Pharmacokinetic analyses

Pharmacokinetic analyses were conducted following US Food and Drug Administration (US FDA) Draft Guidance For Industry On Pharmacokinetics In Patients With Impaired Renal Function (2010). Analyses included all subjects who received at least 1 dose of study drug and had plasma concentration data above the lower limit of quantitation. Details of sample collection and bioanalytical methods are provided in Additional file 1. Pharmacokinetic parameters were calculated using noncompartmental analysis with WinNonlin Professional v6.2.1 (Pharsight Corporation, Cary, NC). Parameters included area under the plasma concentration-time curve (AUC) from time zero extrapolated to infinity (AUC_inf_); AUC from time zero to last measurable concentration (AUC_last_); AUC over the 12 hour dosing interval (AUC_tau_); accumulation ratio (ARAUC_tau_ , based on AUC_tau_ Day 4/ AUC_tau_ Day 1); area under the arterial plasma concentration versus time from beginning to end of dialysis (AUC_d_); maximum observed plasma concentration (C_max_); time of maximum observed plasma concentration (T_max_); and plasma half-life (T_1/2_). Dialysate parameters included amount of drug removed during dialysis for each collection interval (Arem_(t1-t2)_); percentage of total amount of drug recovered in the dialysate (%Arem) calculated as Arem_(0-end)_/dose; and dialysis clearance (CLd; Arem_[0­end]_/AUC_d_).

### Statistical analyses

All statistical analyses were performed using SAS v9.1.3 (SAS Institute Inc, Cary, NC). Pharmacokinetic parameters were summarized using descriptive statistics (n, mean, standard deviation [SD], minimum and maximum values, and percentage coefficient of variance [CV]). Descriptive statistics for T_max_ were summarized using n, median, minimum, and maximum values. Geometric mean and CV values were derived for plasma C_max_, AUC_last_, AUC_tau_, AUC_d_, %Arem, and T_1/2_. Attainment of nalbuphine steady-state was assessed based on visual comparison of trough concentrations. The effect of renal impairment on nalbuphine PK was assessed by analysis of variance (ANOVA) on the natural log transformed PK parameters (AUC and C_max_) on dialysis and non-dialysis days using a general linear mixed effect model and measuring the amount of drug removed in the dialysate.

### Visual analog scale assessment of itch severity

Patients self-reported twice a day their worst daytime and nighttime itch intensity using a visual analog scale (VAS) of 0 (none) to 100 mm (maximal possible intensity) itch score. Patients drew a vertical line between “0” and “100” to denote the worst itching. All VAS values were converted to a scale of 0–10 by dividing the observed value by 10. The average worst VAS score and change from baseline were calculated for each HD patient at each dose level. Baseline VAS score was defined as the average of the values obtained pre-treatment. Data were summarized using descriptive statistics.

### Safety

Safety assessments included the evaluation of adverse events (AEs), clinical laboratory results (serum chemistry, hematology, urinalysis), vital signs (systolic and diastolic blood pressure, pulse rate, respiratory rate, body temperature) and extensive oxygen saturation (SpO_2_) monitoring, 12-lead electrocardiogram (ECG) measurements, and physical examination findings.

## Results

### Patient characteristics

A total of 24 subjects in 2 cohorts were enrolled: 15 HD patients were enrolled in Cohort 1 (12 males and 3 females), of whom 14 completed the study and 1 discontinued; 9 healthy subjects were enrolled in Cohort 2 (7 males and 2 females), of whom 8 completed the study and 1 discontinued. Healthy subjects were matched to HD patients for gender, BMI and age. Patient characteristics are presented in Additional file 1: Table S1.

### Safety

Nalbuphine was well tolerated in all subjects. The most commonly reported treatment emergent AEs (TEAEs) were gastrointestinal and nervous system disorders consistent with the opioid class of drugs. One HD patient discontinued on Day 3 due to a serious AE (SAE) that was considered unlikely to be study drug related. A second HD patient discontinued due to a nonserious, possibly related, Grade 3 report of vertigo after receiving two 240-mg doses; this subject was not replaced. Among healthy subjects, 1 subject discontinued due to a nonserious combined report of Grade 1 gastroesophageal reflux disease, nausea, and vertigo at the 120-mg dose. No deaths were observed in either cohort and there were no apparent treatment-related trends in clinical laboratory assessments, vital sign and SpO_2_ measurements, ECG results, or physical examination findings.

### Pharmacokinetics

**Figure 2:**
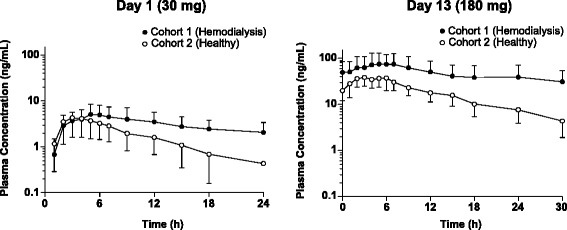
**Plasma concentration of nalbuphine in hemodialysis patients and healthy subjects following a single 30-mg dose on day 1 and a single 180-mg dose on day 13 administered orally as nalbuphine HCl ER tablets.**

Mean plasma concentrations for Day 1 and Day 13 as a function of time for HD patients and healthy subjects are shown in Figure 2. In HD patients, nalbuphine plasma profile was characterized by a slow rise in concentration, reaching a peak within 4–9 hours. For many subjects, plasma profiles were characterized by a double peak pattern, which is suggestive of enterohepatic recirculation. Upon repeated dosing, steady state was attained within 2–3 days, with no significant accumulation in exposure beyond that expected for repeat-dosing (ARAUC_tau_ = 2.7; Table 1). Mean C_max_ ranged between 13 and 83 ng/mL and AUC_tau_ between 118 and 761 ng∙h/mL. Mean plasma half life (T_1/2_) was 10.5 and 14.2 hours following a single 30-mg and repeat 180-mg BID dose, respectively. Exposure (C_max_ and AUC_tau_) increased in a nearly dose proportional fashion over the 30-mg to 180-mg BID dose range: 2-, 4-, and 6-fold increases in dose resulted in approximately 2-, 5-, and 6-fold increases in mean C_max_, and AUC_tau_ (Table 2). Note, data from the 240-mg BID dose are shown for completeness but were not included in the analysis due to the small sample size.

**Table 1:** **Mean pharmacokinetic parameters on day 1 and day 13 following multiple nalbuphine oral doses**

**Parameter**	**Statistics**	**Hemodialysis patients**		**Healthy subjects**	
		**30 mg QD**	**180 mg BID**	**30 mg QD**	**180 mg BID**
		**Day 1**	**Day 13**	**Day 1**	**Day 13**
**AUC**_**inf**_ (ng•h/mL)	N	4	4	7	8
	Mean	142.5	2635.38	49.53	588.40
	SD	33.28	2038.01	30.04	214.08
	CV%	23.4	77.3	60.7	36.4
**AUC**_**last**_ (ng•h/mL)	N	15	9	9	8
	Mean	73.43	1457.74	40.55	529.85
	SD	41.81	1016.26	22.96	179.93
	CV%	56.9	69.7	56.6	34.0
**AUC**_**tau**_ (ng•h/mL)	N	15	9	9	8
	Mean	43.2	760.87	31.53	351.15
	SD	24.97	538.28	16.93	118.21
	CV	57.8	70.7	53.7	33.7
**ARAUC**_**tau**_ **ratio**	Ratio of Mean	2.7	NA	1.6	NA
**C**_**max**_ (ng/mL)	N	15	9	9	8
	Mean	6.28	82.78	5.2	44.21
	SD	3.36	55.81	2.78	14.54
	CV	53.5	67.4	53.5	32.9
**T**_**max**_ (h)	N	15	9	9	8
	Min	1.0	2.0	2.0	2.0
	Median	5.0	5.0	3.0	4.0
	Max	18	7.1	5.0	6.0
**T**_**1/2**_ (h)	N	4	4	7	8
	Mean	10.49	14.23	6.81	8.58
	SD	2.22	3.24	2.79	2.05
	CV	21.1	22.7	41.0	23.9

Subjects were titrated every 3–4 days from 30 mg QD on Day 1 to 30 mg BID then 60 mg BID, 120 mg BID and finally 180 mg BID over a 14-day period. Data shown for Day 1 and Day 13 only.

*Abbreviations*: *ARAUC*_*tau*_ accumulation ratio (mean AUC_tau_ Day 4/Mean AUC_tau_ Day 1), *AUC*_*inf*_ area under plasma concentration-time curve from time zero extrapolated to infinite time, *AUC*_*last*_ area under the plasma concentration-time curve from time zero to the last measureable concentration, *AUC*_*tau*_ area under plasma concentration-time curve over dosing interval (0-12 hr), *BID* twice daily, *C*_*max*_ maximum observed plasma concentration, *CV* coefficient of variation, *ER* extended release, *h* hour, *Max* maximum, *Min* minimum, *n* number of subjects, *NA* not applicable, *QD* once daily, *T*_*max*_ time of maximum observed plasma concentration, *T*_*1/2*_ plasma half life.

**Table 2:** **Mean pharmacokinetic parameters following multiple escalating oral nalbuphine doses in hemodialysis patients**

**Parameter**	**Statistics**	**Non-dialysis days**					**Dialysis days**				
		**30 mg BID**	**60 mg BID**	**120 mg BID**	**180 mg BID**	**240 mg BID**	**30 mg BID**	**60 mg BID**	**120 mg BID**	**180 mg BID**	**240 mg BID**
		**Day 4**	**Day 6**	**Day 9**	**Day 13**	**Day 15**	**Day 3**	**Day 7**	**Day 10**	**Day 12**	**Day 14**
**AUC**_**tau**_ (ng•h/mL)	n	14	10	10	9	3	11	10	10	13	3
	Mean	117.97	221.68	621.79	760.87	769.99	118.56	255.54	582.15	646.06	539.72
	SD	76.41	145.04	415.94	538.28	509.88	74.93	157.81	374.09	433.26	476.19
	CV	64.8	65.4	66.9	70.7	66.2	63.2	61.8	64.3	67.1	88.2
**C**_**max**_ (ng/mL)	n	14	10	10	9	3	11	10	10	13	4
	Mean	13.44	24.78	70.33	82.78	80.47	12.84	27.04	62.51	69.12	63.45
	SD	8.31	17.38	48.81	55.81	51.76	7.71	15.74	40.11	47.20	40.10
	CV	61.8	70.1	69.4	67.4	64.3	60.1	58.2	64.2	68.3	63.2
**T**_**max**_ (h)	n	14	10	10	9	3	11	10	10	13	4
	Min	0	0	3.0	2.0	3.1	2.0	0	0	0	0
	Median	4.0	5.0	6.0	5.0	9.0	4.0	4.0	3.5	3.0	2.0
	Max	9.0	9.0	9.0	7.1	12.0	11.9	11.9	4.0	11.9	4.0
**AUC**_**d**_ (ng•h/mL)	n						11	10	10	9	
	Mean	NA	NA	NA	NA	NA	40.57	86.87	194.95	280.33	NA
	SD						28.14	55.63	136.98	217.42	
	CV%						69.4	64.0	70.3	77.6	
**%Arem**	n						11	10	10	9	
	Mean	NA	NA	NA	NA	NA	0.95	1.07	1.24	1.11	NA
	SD						0.69	0.74	0.91	0.85	
	CV%						73.0	69.2	73.1	76.0	
**CL**_**d**_^**a**^ (L/h)	n						11	10	10	9	
	Mean	NA	NA	NA	NA	NA	6.98	7.33	7.60	7.32	NA
	SD						1.40	1.16	1.30	1.04	
	CV%						20.1	15.8	17.1	14.2	

^a^ Values correspond to 116, 122, 127, and 122 mL/min, respectively.

*Abbreviations*: *%Arem* percentage of total amount of drug removed by hemodialysis, *AUC*_*d*_ area under arterial plasma concentration-time curve from beginning to end of dialysis, *AUC*_*tau*_ area under plasma concentration-time curve over 12 h, *BID* twice daily, *CL*_*d*_ dialysis clearance, *C*_*max*_ maximum observed plasma concentration, *CV* coefficient of variation, *ER* extended release, *h* hour, *n* number of subjects, *NA* not applicable, *QD* once daily, *T*_*max*_ time of maximum observed plasma concentration.

**Figure 3:**
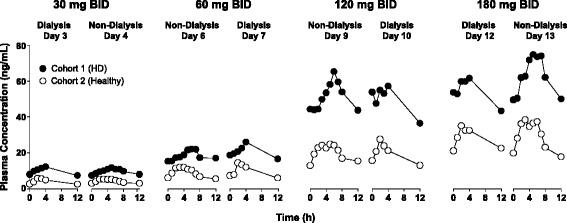
**Plasma concentration of nalbuphine, administered orally as nalbuphine HCl ER tablets, as a function of day and dose.**

In healthy subjects, mean exposure ranged from 5.2 to 44.2 ng/mL for C_max_ and from 31.5 to 351.2 ng∙hr/mL for AUC_tau_ over the 30-mg to 180-mg dose range, with median T_max_ between 2 and 5 hours. As with HD patients, steady state appeared to be attained within 2–3 days of dosing, with a modest accumulation in exposure (ARAUC_tau_ = 1.6). Mean T_1/2_ was 6.8 and 8.6 hours following a single 30-mg and repeat 180-mg BID dose, respectively (Table 1, Additional file 1: Table S2). Exposure in HD patients was significantly higher by 65% (C_max_) and 83% (AUC_tau_) compared to healthy subjects, while T_1/2_ was 1.6-fold longer than in healthy subjects (Additional file 1: Table S3). Overall intersubject variability was high, particularly in HD patients (CV range 54%-71% for C_max_ and AUC_tau_) compared to healthy subjects (CV range 33%-56%). An overlay of nalbuphine plasma concentration profiles as a function of time, dose, and study day for Cohorts 1 and 2 is shown in Figure 3.

### Effect of dialysis on nalbuphine pharmacokinetics

Mean PK parameters for HD patients on dialysis days and non-dialysis days as a function of dose are compared in Table 2. Summary statistics for nalbuphine PK parameters are provided in Table 3. Nalbuphine exposure in HD patients on dialysis days and non-dialysis days was comparable. The geometric mean ratio for dialysis/non-dialysis days (90% confidence interval [CI]) was 98.90 (89.73-109.01) for C_max_ for all doses; and 91.85 (81.02-104.12), 94.51 (83.46-107.03) and 94.64 (82.95-107.99) for AUC_tau_ at the 30, 120 and 180-mg dose levels, respectively. Despite the small number of subjects, the 90% CI for C_max_ and AUC_0-last_ were fully contained within the 80% to 125% confidence limits except at the 60-mg dose, where the upper 90% CI for AUC_tau_ was outside the upper limit. Regardless, the observed difference of 18% is small and was not considered clinically relevant in view of the rather high intersubject variability.

**Table 3:** **Statistical analysis of the effects of hemodialysis on the pharmacokinetics of nalbuphine**

**Parameter**	**Dose (mg)**	**N** ^**a**^	**Geometric means**		**Statistics**	
			**On dialysis (test, T)**	**Non-dialysis (reference, R)**	**GMR (T/R)**	**90% Confidence limit**
**AUC**_**tau**_ (ng•h/mL)	30	11/14	86.46	94.14	91.85	81.02, 104.12
	60	10/10	188.59	159.84	117.99	103.56, 134.43
	120	10/10	418.26	442.56	94.51	83.46, 107.03
	180	13/9	567.05	599.15	94.64	82.95, 107.99
**C**_**max**_ (ng/mL)	All doses	15/14	31.04	31.39	98.90	89.73, 109.01

^a^Number of patients on dialysis/non-dialysis days.

*Abbreviations*: *AUC*_*tau*_ area under the plasma concentration-time curve over the dosing interval, *CI* confidence interval, *C*_*max*_ maximum observed plasma concentration, *h* hour, *GMR* geometric mean ratio.

Analysis of nalbuphine concentration in dialysate indicated that 0.95%-1.24% of the dose was removed during a standard high-flux 3–4 hour hemodialysis session over the dosing range (%Arem; Table 2). Clearance during dialysis (CL_d_), calculated based on arterial blood sampling from the dialyzer port during dialysis, was 7–7.6 L/kg (or 116–127 mL/min) and approximated the creatinine clearance in subjects with normal kidney function (>90 mL/min).

### VAS assessment of itch severity

**Figure 4:**
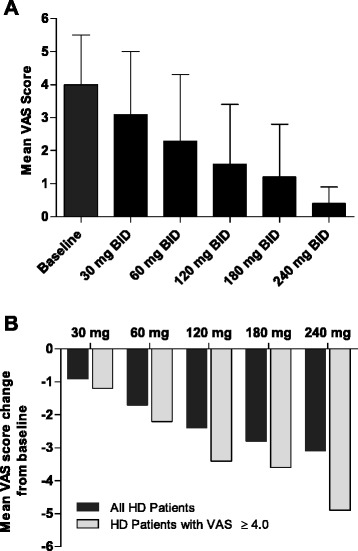
**Comparison of mean VAS score of itch severity (A) and change from baseline (B) as a function of nalbuphine HCl ER dose.**

The impact of nalbuphine HCl ER tablets on uremic pruritus was explored in HD patients who self-reported itch intensity using a VAS score. Nalbuphine suppressed itch in a dose-dependent manner in 12/14 patients, reducing itch from a mean VAS score of 4.0 (range, 1.3-6.6) to 1.2 and 0.4 at 180 mg and 240 mg, respectively (Table 4, Figure 4A). Itch intensity in HD patients is reported to fluctuate and appears to be cyclical in some patients [1]. However, patients with a baseline VAS above 4 (40 mm) are reported to have a more persistent itch (daily or nearly daily) and changes in VAS of at least 20% in either direction are considered indicative of a change in patient-rated pruritus severity [1]. Of the 14 patients assessed in this study, 8 had VAS score ≥4.0 (mean, 5.1; range, 4.2-6.6). Subgroup analysis of these patients showed a more pronounced change compared to all patients treated, with a mean change from baseline of –1.2, −2.2, –3.4, −3.6 and −4.9 at the 30-, 60-, 120-, 180- and 240-mg BID doses, respectively, with the largest incremental changes occurring between 60 mg and 120 mg BID (Table 4, Figure 4B).

**Table 4:** **Mean VAS score as a function of nalbuphine oral dose in hemodialysis patients**

**Dose**	**Statistics**	**VAS score**		**Change from baseline**	
		**All patients**	**Patients with VAS ≥ 4.0**	**All patients**	**Patients with VAS ≥ 4.0**
**Baseline**	N	14	8	--	--
	Mean (SD)	4.0 (1.5)	5.1 (0.8)		
	Median	4.4	4.9		
	Min, Max	1.3, 6.6	4.2, 6.6		
**30 mg BID**	N	14	8	14	8
	Mean (SD)	3.1 (1.9)	3.9 (1.9)	−0.9 (1.3)	−1.2 (1.5)
	Median	2.8	3.2	−0.5	−1.7
	Min, Max	0.4, 6.7	1.4, 6.7	−3.2, 0.8	−3.2, 0.8
**60 mg BID**	N	14	8	14	8
	Mean (SD)	2.3 (2.0)	2.9 (2.2)	−1.7 (1.8)	−2.2 (1.8)
	Median	1.9	2.8	−1.5	−1.8
	Min, Max	0.1, 6.2	0.1, 6.2	−4.3, 1.2	−4.3, 0.8
**120 mg BID**	N	14	8	14	8
	Mean (SD)	1.6 (1.8)	1.7 (2.1)	−2.4 (1.9)	−3.4 (1.9)
	Median	0.8	0.9	−2.6	−4.0
	Min, Max	0.0. 6.1	0.0, 6.1	−5.5, 0.9	−5.5, 0.7
**180 mg BID**	N	13	7	13	7
	Mean (SD)	1.2 (1.6)	1.6 (2.0)	−2.8 (1.7)	−3.6 (1.8)
	Median	0.8	0.8	−2.6	−3.9
	Min, Max	0.0, 5.8	0.1, 5.8	−5.1, 0.4	−5.1, 0.4
**240 mg BID**	N	4	2	4	2
	Mean (SD)	0.4 (0.5)	0.7 (0.6)	−3.1 (2.1)	−4.9 (0.8)
	Median	0.3	0.7	−2.8	−4.9
	Min, Max	0.0, 1.2	0.3, 1.2	−5.5, −1.3	−5.5, −4.3

*Abbreviations*: *BID* twice daily, *ER* extended release, *SD* standard deviation, *VAS* visual analog scale.

## Discussion

Pharmacokinetics of nalbuphine following oral administration of nalbuphine HCl ER tablets up to 15 days were assessed in HD patients with pruritus compared to matched healthy control subjects. A dose-escalation study design was selected to mimic nalbuphine use in uremic pruritus patients in subsequent clinical efficacy studies where patients would start at a low dose to minimize common opioid AEs such as nausea and vomiting and allow development of some tolerance to these particular AEs. Ultimately, nalbuphine would be titrated to effect, as is standard in opioid treatment, and a washout period between doses would counter the intent of titration, hence the continuous escalation from 30 mg to 240 mg in this study. Nalbuphine is a low molecular weight, water soluble molecule, with low protein binding (~50%) and a large volume of distribution (315.5 L) [28,29]. Nalbuphine is a high extraction (perfusion-limited) drug [29], predominantly hepatically cleared in the feces [30,31]. In HD patients, changes in hepatic blood flow as well as diffusion of the drug through the dialysis membrane have the potential to affect nalbuphine exposure, although the large volume of distribution is expected to offset the dialysis effect. In this study we show that nalbuphine exposure in HD patients on non-dialysis and dialysis days was comparable over a 6-fold dose range with only 1% of the dose being removed by dialysis. There was no significant drug accumulation, beyond that expected for repeat dosing. Collectively, these findings indicate that no dose adjustment around dialysis treatment is needed.

Following repeat dosing, nalbuphine exposure increased in a nearly dose-proportional fashion, reaching steady state within 2-3 days at all dose levels suggesting that additional accumulation due to more prolonged exposure is unlikely. Exposure was significantly higher in HD patients than healthy subjects (83% and 65% increase in AUC_tau_ and C_max_), most likely due to the longer half-life in HD patients.

Nalbuphine is metabolized and cleared by the liver thus both liver function and genetic differences in drug metabolizing enzymes and transporters among race groups could potentially result in variability in pharmacokinetics. For the marketed *Nalbuphine HCl for Injection*, dose reduction is recommended in patients with hepatic dysfunction [18] since higher exposures are expected. In this study, only subjects with normal to mild impaired liver function were included as the effect of significant co-existing liver disease on nalbuphine safety and exposure in HD patients is not yet understood. It is also worth noting that there were more blacks or African Americans enrolled in the HD group (73%) compared to the healthy subjects (44%). Whether race played a role in the pharmacokinetic differentiation between HD patients and healthy subjects cannot be gauged from this study due to the small number of subjects. However, it does underscore the need for evaluation of the role of polymorphisms in metabolic pathways on nalbuphine exposure in future clinical studies.

Assessments of AEs, clinical laboratory results, vital signs, oxygen saturation, and physical examination findings demonstrated that nalbuphine HCl ER oral tablets were safe and well-tolerated up to the 240-mg BID dose tested in HD patients. Moreover, while this study was not specifically designed to assess effects on uremic pruritus, discernible reductions in VAS measures of itch severity did appear to be a function of increasing nalbuphine dose in HD patients despite the limited sample size.

## Conclusions

In summary, nalbuphine administered as oral nalbuphine HCl ER tablets was safe and well-tolerated in HD patients. Nalbuphine is not extracted by dialysis. Exploratory investigations suggest that nalbuphine HCl ER tablets may be effective in reducing pruritus in HD patients, with particular benefit at doses of 60 mg BID or higher. Well-controlled clinical efficacy studies will be conducted to establish the longitudinal effect of treatment with nalbuphine HCl ER tablets on uremic pruritus and assess its long term safety.

## Additional files

Additional file 1: Table S1. Patient Demographics and Baseline Characteristics. **Table S2.** Mean Pharmacokinetic Parameters Following Multiple Escalating Oral Doses of Nalbuphine HCl ER Tablets in Cohort 2 Healthy Subjects on Non-Dialysis and Dialysis Days. **Table S3.** Statistical Analysis of the Pharmacokinetics of Nalbuphine in Hemodialysis Patients Versus Healthy Subjects.
