# Supplementary material for: Pharmacokinetics of nalbuphine hydrochloride extended release tablets in hemodialysis patients with exploratory effect on pruritus
Source: BMC Nephrol. 2015 Apr 8;16:47. doi: 10.1186/s12882-015-0043-3 (PMC4392787; doi:10.1186/s12882-015-0043-3)
Supplement: Additional file 1: Table S1. — Patient Demographics and Baseline Characteristics. Table S2. Mean Pharmacokinetic Parameters Following Multiple Escalating Oral Doses of Nalbuphine HCl ER Tablets in Cohort 2 Healthy Subjects on Non-Dialysis and Dialysis Days. Table S3. Statistical Analysis of the Pharmacokinetics of Nalbuphine in Hemodialysis Patients Versus Healthy Subjects. [file 12882_2015_43_MOESM1_ESM.docx]

SUPPLEMENTARY METHODS

***Hemodialysis Conditions***

Hemodialysis was conducted under conditions prescribed during the patients’ conventional hemodialysis sessions. Briefly, dialysis was conducted over 3-3.5 hours using a Fresenius (K) machine and a high flux dialyzer, polysulfone (Fresenius Optiflux 180 NR) hemofilter. Venous access was obtained by cannulation of the patient’s hemodialysis arteriovenous ﬁstula or polytetraﬂuoroethylene graft. The blood flow rate was maintained between 300-450 mL/min. Dialysate was pumped countercurrent to blood using a sodium bicarbonate buffer (Minntech Renal Systems: Centrisol Bicarbonate Concentrate Powder (part B) MB-330-25 45X) with a flow rate between 600 and 700 mL/min. Heparin was infused through a prehemoﬁlter port to control the activated clotting time. For each HD session, the average blood flow rate, average dialysate flow rate, duration of dialysis, and the make and model of the dialyzer was recorded.

***Pharmacokinetics Analyses***
Pharmacokinetic parameters were derived from plasma and dialysate nalbuphine concentration versus time data using noncompartmental analysis with WinNonlin Professional v6.2.1 (Pharsight Corporation, Cary, NC). Actual post-dose sampling times were used to derive all parameters. All concentration values below the lower limit of quantitation were set to zero.

***Pharmacokinetic Sampling***

Blood samples (2 mL) were collected on study Day 1, and Day 13 or Day 15 (Cohort 1, Group 4 only) pre-morning dose, and then 1, 2, 3, 4, 5, 6, 7, 9, 12, 15, 18, 24, and 30 hours post the morning dose. Additional samples were collected for subjects in Cohort 1, Group 4 at 36, 48, and 54 hour time points. On study Days 4, 6, and 9 blood samples were collected pre-morning dose, and then post dose at 1, 2, 3, 4, 5, 6, 7, 9, and 12 hours (pre-evening second dose). On days corresponding to hemodialysis days (Days 3, 7, 10, and 12) blood samples were collected at pre‑morning dose and then at 1, 2, 3, 4 and 12 hours post‑dose (prior to the evening dose administration). For Cohort 1 subjects, simultaneous arterial and venous blood samples were obtained during dialysis at t=0 (immediately before dialysis) and then at 1, 2, 3 hours during dialysis and at the end of the dialysis session. Trough blood samples were collected on Days 2, 3, 5, 7, 8, 10, and 11 prior to morning and/or evening dose administrations. Additional samples were collected on Days 6, 9, and 13 from subjects in Cohort 1, Group 4.

Dialysate samples were collected from Cohort 1, Group 1-3 during dialysis over 1‑hour intervals from 0-1, 1-2, 2-3, and from 3 hours until dialysis was completed on each dialysis day while on treatment.

All blood samples were collected into sodium EDTA (2-8°C) tubes and centrifuged at approximately 1500 g for 10 minutes, and the resulting plasma was divided into 2 aliquots and stored in polypropylene cryotubes at −70°C, until analyzed. Dialysate samples were divided into aliquots and stored under identical conditions to those of the plasma samples.

Note that urine could not be collected from HD patients as they were anuric.

***Nalbuphine Bioanalytical Assay***Nalbuphine concentration in plasma and dialysate samples was assayed using a validated reverse-phase high-performance liquid chromatography with tandem mass spectrometric detection (LC-MS/MS) method with deuterated (D3) nalbuphine as an internal standard. Nalbuphine and the internal standard were extracted from plasma by protein precipitation with acetonitrile followed by centrifugation or by liquid-liquid extraction for dialysate. The supernatant was analyzed by high‑performance liquid chromatography (HPLC) with selective detection of nalbuphine parent to product ion mass transition of m/z 358.2 to 343.2. Concentrations were calculated from an 8-point calibration curve using a weighting factor of 1/x2 quadratic equation, and acceptance criteria were based on quality control sample and incurred sample reproducibility standard versus the corresponding nominal concentrations.

For plasma samples, the assay range was 0.5 to 50.0 ng/mL with a lower limit of quantification of 0.5 ng/mL. Accuracy and precision of the assay ranged between -2% to 0% and 5.4% to 5.8%, respectively. For dialysate samples, the assay range was 0.3 to 30.0 ng/mL with a lower limit of quantification of 0.3 ng/mL.

**SUPPLEMENTARY DATA TABLES**

Table S1: Patient Demographics and Baseline Characteristics

| **Parameters** | Hemodialysis Patients (N = 15) | Healthy Subjects (N = 9) | **Overall**  **(N = 24)** |
| --- | --- | --- | --- |
| **Age** (years) |  |  |  |
| Mean (SD) | 46.6 (10.1) | 50.1 (5.1) | 47.9 (8.6) |
| Minimum, maximum | 25, 61 | 39, 57 | 25, 61 |
| **Gender**, n (%) |  |  |  |
| Female | 3 (20.0) | 2 (22.2) | 5 (20.8) |
| Male | 12 (80.0) | 7 (77.8) | 19 (79.2) |
| **Race**, n (%) |  |  |  |
| White | 4 (26.7) | 5 (55.6) | 9 (37.5) |
| Black or African American | 11 (73.3) | 4 (44.4) | 15 (62.5) |
| **Ethnicity**, n (%) |  |  |  |
| Hispanic or Latino | 1 (6.7) | 0 | 1 (4.2) |
| Not Hispanic or Latino | 14 (93.3) | 9 (100.0) | 23 (95.8) |
| **Height** (cm) |  |  |  |
| Mean (SD) | 172.6 (11.4) | 173.1 (8.3) | 172.8 (10.2) |
| Minimum, maximum | 148.2, 191.7 | 159.4, 186.5 | 148.2, 191.7 |
| **Weight** (kg) |  |  |  |
| Mean (SD) | 88.6 (19.0) | 86.5 (7.6) | 87.8 (15.5) |
| Minimum, maximum | 52.0, 120.9 | 75.5, 96.1 | 52.0, 120.9 |
| **Body mass index** (kg/m^2^) |  |  |  |
| Mean (SD) | 29.5 (4.6) | 28.9 (2.2) | 29.3 (3.9) |
| Minimum, maximum | 21.0, 40.7 | 25.2, 31.1 | 21.0, 40.7 |
| **Kt/V** |  |  |  |
| Median | 1.5 | NA | NA |
| Minimum, maximum | 1.4, 2.1 |  |  |
| Abbreviation: SD, standard deviation. | | | |

Table S2: Mean Pharmacokinetic Parameters Following Multiple Escalating Oral Doses of Nalbuphine HCl ER Tablets in Cohort 2 Healthy Subjects on Days Corresponding to Cohort 1 Non-Dialysis and Dialysis Days

|  |  | **Days Corresponding to Cohort 1**  **Non-Dialysis Days** | | | | **Days Corresponding to Cohort 1**  **Dialysis Days** | | | |
| --- | --- | --- | --- | --- | --- | --- | --- | --- | --- |
| **Parameter** | **Statistics** | **30 mg BID** | **60 mg BID** | **120 mg BID** | **180 mg BID** | **30 mg BID** | **60 mg BID** | **120 mg BID** | **180 mg BID** |
|  |  | **Day 4** | **Day 6** | **Day 9** | **Day 13** | **Day 3** | **Day 7** | **Day 10** | **Day 12** |
| **AUC_tau_** (ng•h/mL) | n | 9 | 9 | 9 | 8 | 9 | 9 | 9 | 8 |
|  | Mean | 50.88 | 106.11 | 240.37 | 351.15 | 48.05 | 117.44 | 226.24 | 341.68 |
|  | SD | 27.54 | 50.49 | 93.68 | 118.21 | 24.41 | 54.14 | 89.52 | 117.08 |
|  | CV | 54.1 | 47.6 | 39 | 33.7 | 50.8 | 46.1 | 39.6 | 34.3 |
| **C_max_**  (ng/mL) | n | 9 | 9 | 9 | 8 | 9 | 9 | 9 | 8 |
|  | Mean | 6.45 | 13.46 | 28 | 44.21 | 6.33 | 15.32 | 29.19 | 37.14 |
|  | SD | 3.58 | 6.43 | 11.49 | 14.54 | 3.73 | 8.26 | 12.09 | 12.48 |
|  | CV | 55.5 | 47.8 | 41 | 32.9 | 58.9 | 53.9 | 41.4 | 33.6 |
| **T_max_**  (h) | N | 9 | 9 | 9 | 8 | 9 | 9 | 9 | 8 |
|  | Min | 2.0 | 2.0 | 3.0 | 2.0 | 2.0 | 1.0 | 1.0 | 1.0 |
|  | Median | 2.0 | 3.0 | 5.0 | 4.0 | 3.0 | 2.0 | 2.0 | 2.5 |
|  | Max | 6.0 | 6.0 | 9.0 | 6.0 | 4.0 | 4.0 | 3.0 | 4.0 |
| Abbreviations: AUC_tau_, area under plasma concentration-time curve over 12h; BID, twice daily; C_max_, maximum observed plasma concentration; CV, coefficient of variation; ER, extended release; h, hour; n, number of subjects; T_max_, time of maximum observed plasma concentration. | | | | | | | | | |

Table S3: Statistical Analysis of the Pharmacokinetics of Nalbuphine in Hemodialysis Patients Versus Healthy Subjects

| **Parameter** | **Dose**  **(mg)** | **N^a^** | **Geometric Means** | | **Statistics** | |
| --- | --- | --- | --- | --- | --- | --- |
|  |  |  | Hemodialysis (HD) | Healthy  (H) | GMR (HD/H) | 90% Confidence Limit |
| **AUC_tau_** (ng•h/mL) | All doses | 15/9 | 273.38 | 149.23 | 183.19 | 152.68, 219.79 |
| **C_max_** (ng/mL) | All doses | 15/9 | 29.41 | 17.79 | 165.37 | 138.72, 197.13 |
| ^a^ Number of hemodialysis patients/healthy subjects  Abbreviations: AUC_tau_, area under the plasma concentration-time curve over the dosing interval; CI, confidence interval; C_max_, maximum observed plasma concentration, h, hour; GMR, geometric mean ratio. | | | | | | |
